# Photocatalytic degradation of dimethoate in Bok choy using cerium-doped nano titanium dioxide

**DOI:** 10.1371/journal.pone.0197560

**Published:** 2018-05-17

**Authors:** Xiangying Liu, Yu Li, Xuguo Zhou, Kun Luo, Lifeng Hu, Kailin Liu, Lianyang Bai

**Affiliations:** 1 College of Plant Protection, Hunan Agricultural University, Changsha, Hunan province, PR China; 2 Department of Entomology, University of Kentucky, Lexington, Kentucky, United States of America; 3 Collaborative Innovation Center of Farmland Weeds Control, Loudi, Hunan province, PR China; 4 Biotechnology Research Center, Hunan Academy of Agricultural Sciences, Changsha, Hunan province, PR China; Institute of Materials Science, GERMANY

## Abstract

Dimethoate, a systemic insecticide, has been used extensively in vegetable production. Insecticide residues in treated vegetables, however, pose a potential risk to consumers. Photocatalytic degradation is a new alternative to managing pesticide residues. In this study, the degradation of dimethoate in Bok choy was investigated under the field conditions using cerium-doped nano titanium dioxide (TiO_2_/Ce) hydrosol as a photocatalyst. The results show that TiO_2_/Ce hydrosol can accelerate the degradation of dimethoate in Bok choy. Specifically, the application of TiO_2_/Ce hydrosol significantly increased the reactive oxygen species (ROS) contents in the treated Bok choy, which speeds up the degradation of dimethoate. Ultra-performance liquid chromatography coupled with mass spectrometry (UPLC-MS) analysis detected three major degradation products, including omethoate, O,O,S-trimethyl thiophosphorothioate, and 1,2-Bis (acetyl-N-methyl-) methane disulfide. Two potential photodegradation pathways have been proposed based on the intermediate products. To understand the relationship between photodegradation and the molecular structure of target insecticides, we investigated the bond length, Mulliken atomic charge and frontier electron density of dimethoate using *ab initio* quantum analysis. These results suggest the P = S, P-S and S-C of dimethoate are the initiation sites for the photocatalytic reaction in Bok choy, which is consistent with our empirical data.

## Introduction

Bok choy, a major Brassica vegetable, is cultivated widely throughout the world. Reducing prostate cancer and breast cancer risks are some of the health benefits from Bok choy consumption [1, 2]. During its production, dimethoate (IUPAC name: O, O-dimethyl S-methyl-carbamoylmethyl phosphorodithioate), an organophosphate insecticide and acaricide, has been used extensively to control arthropod pests. The mode of action of dimethoate is to inhibit the activity of cholinesterase, an enzyme essential for normal functioning of the nerve systems of humans, other vertebrates and invertebrates, including insects. Dimethoate can induce neurologic disorders [3, 4], decrease reproductivity [5,6], damage DNA [7, 8] and cause other histopathological changes [9, 10]. Although dimethoate is acutely toxic and categorized as moderately hazardous [11], it is highly toxic to a common carp, *Cyprinus carpio* (Linn.), and severely affects their physiology and behaviour [12]. In addition, residues of dimethoate and its derivatives have been detected in fruits, vegetables, even cow milk. In China, the maximum dimethoate residue limit of 0.2 to 1 mg/kg for vegetables has been established jointly by the Ministry of Agriculture and National Family Planning Council. It is of great importance to limit dimethoate residue in vegetables and fruits to minimize its risks to environment and human health.

Traditionally, physical, chemical, and biological methods have been used to eliminate dimethoate residuals. However, physical degradation methods mainly aim at residues on the surface of vegetables and fruits. Chemical degradation can be effective, however, incurs a higher cost. Also, the toxicity of secondary metabolites needs further investigation. Biological degradation is mainly through dilution, with bacteria or fungi as medium. The disadvantages, however, are associated with a higher dilution rate, fluctuation of services and secondary pollution [13]. Advanced oxidation processes (AOPs), a procedure based on the photo-excitation of a semiconductor, has drawn more attentions in recent years. Specifically, under the ultraviolet light or sunlight, photocatalytic reaction may produce negative electron (e^−^) in the conduction band and positive hole (h^+^) in the valance band of a semiconductor. e^−^ and h^+^ are powerful reductive and oxidizing agents. Then oxidation-reduction reaction was induced by e^−^ and h^+^ [14,15]. Holes oxidize H_2_O on the surface of semiconductors and •OH is photogenerated. O_2_ on the surface of semiconductors traps electron and O_2_^•-^ and O_2_^2-^ are formed. These reactive oxygen species (ROS) (e.g. O_2_^•-^, O_2_^2-^ and •OH) exhibit high oxidative activity for organic compounds. They can readily cleave C-C bond, lead to a partial or total decomposition, and mineralize into CO_2_, H_2_O and inorganic ion (e.g. Cl^-^, NO_3_^-^, SO_4_^2-^) [16, 17]. Particularly, TiO_2_ has been used extensively among semiconductors. Its application involves disinfection [18], environmental purification [19], health care and so on [20]. The main advantages of TiO_2_ photocatalysts are the immunity, catalytic stability, resistance to photocorrosion, low cost and non-toxicity [21, 22]. High photocatalytic activity could yield high mineralization, and enhanced oxidation process is considered a promising technology to resolve pesticide residual issues. However, the photocatalytic activity of TiO_2_ is much higher under the ultraviolet light relative to natural light. To extend the optical absorbance edge into the visible region and to enhance the photocatalytic activity, surface modifications are required, e.g., doping TiO_2_ with Ce, C, S, N, and Ag [23, 24]. Among the multi-doped TiO_2_, Ce doped TiO_2_ showed higher visible light photocatalytic activity [25, 26].

Here, we investigated the photocatalytic degradation of dimethoate with TiO_2_/Ce hydrosol as the catalytic system under the field condition. To reveal the catalytic capacity of TiO_2_/Ce hydrosol, we determined the degradation efficiency of dimethoate and measured the content of ROS in Bok choy, we then carried out UPLC-MS to identify the degradation products. Based on the detected intermediate products, we proposed the potential photodegradation pathway of dimethoate in TiO_2_/Ce system. Finally, the quantum chemistry analyses predict degradation products and the molecular mechanism of photodegradation of dimethoate.

## Materials and methods

### Chemicals and reagents

Nano TiO_2_/Ce hydrosol was donated by Panzhixhua Iron and Steel Research Institute (Sichuan province, China), nano titanium dioxide exists in anatase forms, and Ce doping with a concentration of 0.6% in molar ratio. Dimethoate standard was obtained from Dr. Ehrenstorfer (Augsburg, Germany) with the purity of 99.9%. Commercial dimethoate 40% emulsifiable concentrate (EC) was purchased from Hunan Haili Changde Co., Ltd. (Hunan province, China). The chromatographically pure acetone and methanol were obtained from Tedia Company Inc. (USA). Plant reactive oxygen species (ROS) ELISA Kit (HG55780, Tsz Biosciences, San Francisco, USA). All other reagents were supplied from Sinopharm Chemical Reagent Co., Ltd (Shanghai, China).

### Field trials

TiO_2_/Ce hydrosol-mediated solar photodegradation of commercial dimethoate was conducted at the Research and Training Station of Hunan Agricultural University, Hunan, China. Soil in this region is alluvial, with soil organic matter between 0–20 cm of 10–30 mg/kg and pH range of 4.5–6.5. Here, the experiment plot was 20 m^2^ with a 30 cm buffer area left in-between two plots. Seeds of Bok choy variety “ChuanShan (103)” (HSBC seed co., LTD, Hunan province, China) were directly sowed in the plots with a dosage of 25 kg/ha. The nutrient and water management was employed using conventional agricultural practice. After 20 d, commercial dimethoate at 600 g a.i. /ha was sprayed onto Bok choy leaves with an electric sprayer (type of 3WBD-18, Shijiazhuang Prandi Electromechanical Instrument Co. Ltd, China). Dauterman reported that the dimethoate can rapidly penetrate into the plant leaves, and is quickly absorbed both on the surface and inside the leaves after application [27]. So TiO_2_/Ce hydrosol at 2400 g a.i. /ha was applied to the same leaves after 1 h of dimethoate application. Bok choy leaves without TiO_2_/Ce hydrosol served as the blank control. All treatments were carried out in a randomized design with three biological replications. By five-point sampling method, each 50 Bok choy plants were transported to the laboratory within 2 h of collection, and stored at 4 °C in the refrigerator after 0, 0.5, 1, 2, 3, 4, 5, 6, 7, 8, 9, and 10 d.

### Sample preparation

Dimethoate 40% EC was applied onto Bok choy leaves. Due to dimethoate’s rapid penetration into the plant leaves [27], Bok choy samples were not washed before they were shredded and mixed in a blender. First, 10 g shredded Bok choy were put into a 100 mL glass centrifuge tube containing 50 mL acetonitrile, 4 g anhydrous magnesium sulfate and 6 g sodium chloride for extraction, and followed by homogenization for 2 min at 16000 rpm, then centrifuged for 5 min at 6000 rpm. The resultant 10 mL supernatant was evaporated to dryness by a rotary evaporator at 40 °C, and resuspended in 2 mL acetone. The extracts were then transferred to Florisil solid-phase extraction (SPE) tube (ANPEL laboratory Technologies Inc, Shanghai, China) which was previously washed with 3 mL acetone and 3 mL acetonitrile, subsequently eluted by 4 mL acetone and 10 mL acetonitrile. The eluent was collected and dried by a rotary evaporator at 40 °C, and resuspended in 2 mL acetone for GC analysis and 2 mL methanol for UPLC-MS analysis. Prior to injection, samples were filtered through a 0.22 μm membrane filter.

### Gas chromatography (GC) analysis

The amount of dimethoate residue in Bok choy was determined by GC with a flame photometry detector (FPD) (Shimadzu GC-2010). The detector was linked to a data system for acquisition and calculation. The RTX-5 capillary column was 30 m × 0.25 mm I.D., 0.25 μm film thickness and was employed with nitrogen as carrier gas. The following chromatographic conditions were adopted according to the results of preliminary experiments obtained by injecting directly standard solutions of dimethoate into the chromatographic column. The injector temperature was 220 °C. The injection volume was 1 μL and the split ratio was 5:1. The FPD temperature was 210 °C. The column volume was 1 mL/min. The column temperature was programmed at 120 °C, then it was increased at 20 °C/min rate from 120 °C to 200 °C, and finally it was kept at 200 °C for 5 min. The retention time of dimethoate was 9.3 min under the conditions. The standard curves were obtained based on the peak areas and its corresponding solution concentrations, including 0.01, 0.02, 0.05, 0.1, 0.5, 1.0, 2.0, 5, 10, 50, 100 mg/L. The percent recovery of dimethoate extracted from Bok choy was determined by adding standard dimethoate to blank Bok choy samples at 0.1, 1, 10 mg/kg.

### Reactive oxygen species (ROS) assay

After 1, 2, 3, 4 and 5 d of photocatalytic degradation treatments, Bok choy plants were collected from the field, respectively, to measure the ROS contents using an enzyme-linked immunoassay (ELISA) method. 1 g of minced Bok choy was placed in a centrifuge tube and soaked in ice-water. 9 mL of 10 mmol/L phosphate buffer (pH7.2–7.4) was then added to the tube, homogenized for 2 min under ice-water condition, and centrifuged for 5 min at 4500 rpm at 4 °C. The supernatant was collected to measure the ROS contents following the manufacturer’s protocol of plant reactive oxygen species ELISA Kit (HG55780, Tsz Biosciences, San Francisco, USA).

### UPLC-MS analysis

To detect the degradation products of dimethoate, Bok choy samples were collected for the analysis after treatment 1 d. Analysis was performed by Waters Acquity UPLC^®^ system separations module coupled with a Waters TQD mass spectrometer (Waters, Milford, MA, USA) on a reverse-phase Waters BEH C18 UPLC column (210 × 100 mm, 1.7 μm). The injection volume was 5 μL. The column temperature was kept at 40 °C with a flow rate of 0.25 mL/min. Mobile phases consisted of water (A), methanol (B), each containing 5% ammonium acetate water (5 mmol/mL) (C). The gradient of mobile phases programmed: 5% C hold for the overall process. 90% A and 5% B in 1 min, hold for 1 min, next a linear gradient of B from 5% to 47.5% in 1.5 min, hold for 0.5 min, afterwards a linear gradient of B from 47.5% to 95% in 1min, hold for 3.5 min, then a return to the initial conditions in 2 min before next injection.

An electrospray ionization (ESI)—mass spectrometry (MS) method was carried out for degradation products identification in positive ion mode. The collision gas was argon, and the desolvation gas was nitrogen at a flow rate of 750 L/h. A capillary and voltage cone voltage were 2 kV and 35 V, respectively. The temperatures of desolvation and a source were set at 350 °C and 120 °C, respectively. Under the daughter scan mode with collision gas flow of 0.12 mL/min and collision energy of 20 V, the mass spectrums were acquired ranging from *m*/*z* 50 to 250 with the initial and final retention time set to 0.0 min and 8.0 min, respectively.

### RHF/STO-3G calculation

The quantum chemistry analysis was carried out using Gaussview 3.08 and Hyperchem Release 7.0 package. The optimal geometry conformation and the lowest energy of dimethoate molecule were predicted using RHF/STO-3G. The bond length, the atomic charge and the frontier electron density of the highest occupied molecular orbital (FED_HOMO_^2^) were calculated using Gaussview 3.08 package as well. Chart of the total charge density and the frontier orbital density (HOMO) of dimethoate was calculated using Hyperchem Release 7.0 program package.

### Data analysis

All experiments were carried out twice independently with three replications. To assess the effect of ROS content on TiO_2_/Ce photocatalytic degradation in Bok choy, one way ANOVA was used to compare the ROS content in Bok choy between with and without TiO_2_/Ce under different sampling time. Means were compared with LSD tests at *P* < 0.05. SPSS version 20.0 (SPSS Inc., USA) was used for statistical analyses. UPLC-MS detection data, including the abundance, retention time-m/z pairs, and ion intensity, were analyzed by MassLynx V4.1 software (Waters Corp., USA).

## Results

### Degradation kinetics of dimethoate in Bok choy

The standard curve for dimethoate degradation using GC analysis was y = 2117726 x—1184948 (R^2^ = 0.9968), which showed a linear correlation between the peak area (y) and dimethoate concentration (x) between 0.01 and 100 mg/L. The average recovery of dimethoate ranged from 95 to 117% in Bok choy. The value of limit of detection (LOD) and limit of quantification (LOQ) were 0.007 and 0.024 μg/kg, respectively.

**Fig 1:**
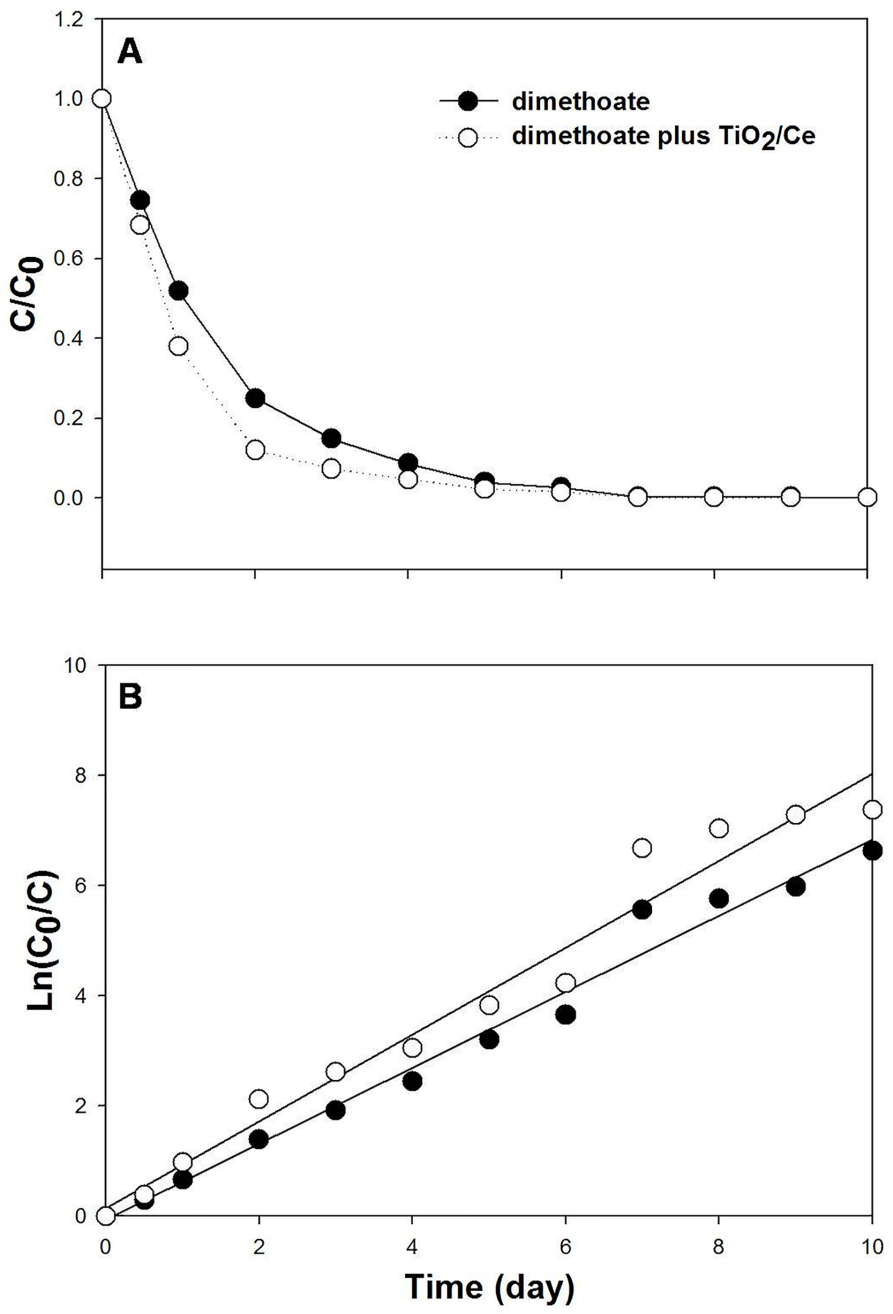
Degradation dynamics of dimethoate in Bok choy by TiO_2_/Ce. The residue change of dimethoate in Bok choy (**A**: original data; **B**: log transferred data). C_0_ and C represent the initial and reacting (time = t) residues of dimethoate in Bok choy, respectively. The C_0_ of dimethoate residual concentrations of 600 g a.i. /ha for time zero was 15.859 mg/kg.

**Fig 2:**
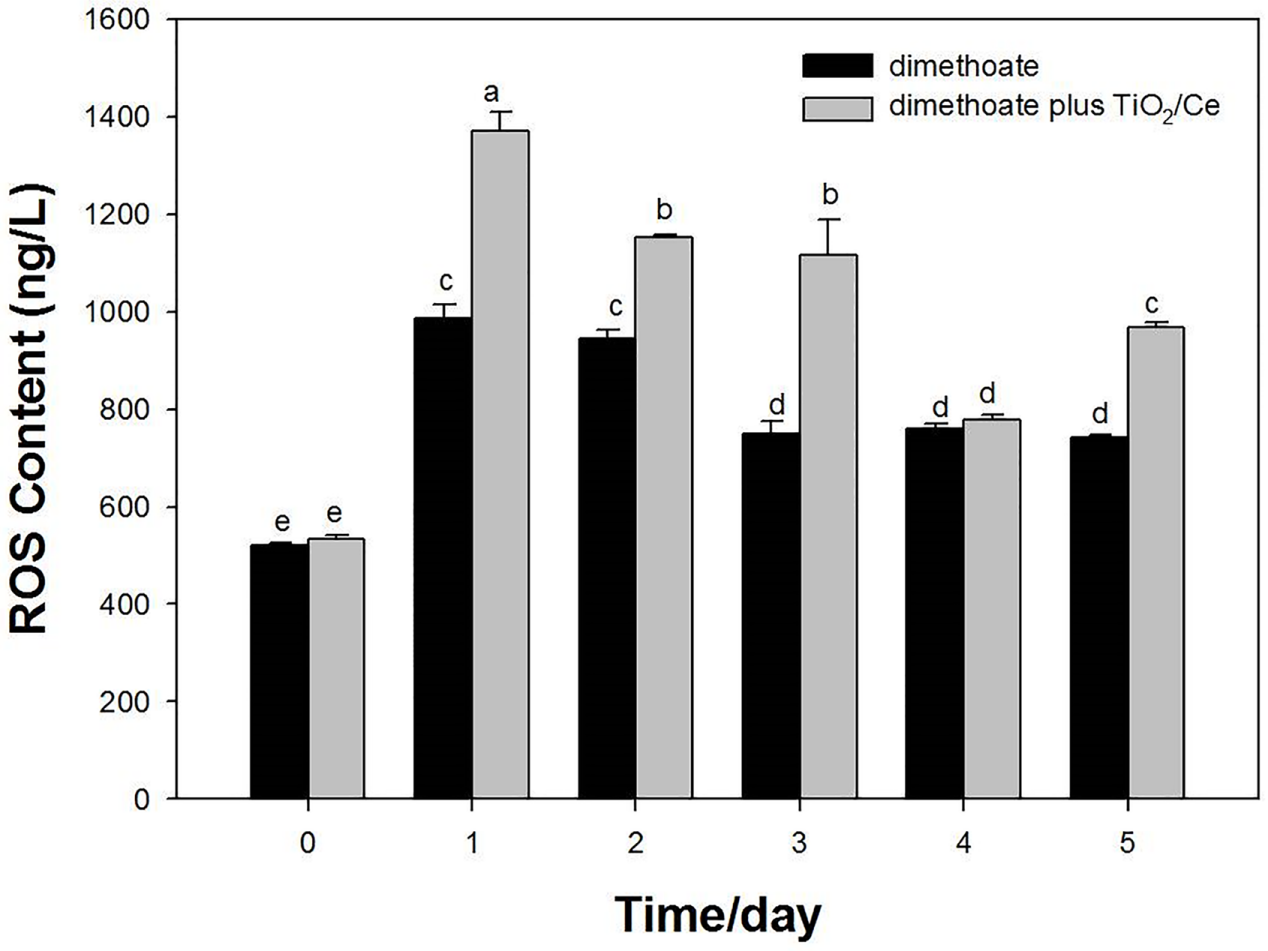
ROS content in Bok choy with and without TiO_2_/Ce treatment. LSD test declares the differences between means (*P* < 0.05). Different letters indicate significant differences among treatments.

With TiO_2_/Ce hydrosol, dimethoate residual was significant reduced in comparison to the controls, especially for 0.5, 1, 2 and 3 d (Fig 1A). The digestion dynamic equation for dimethoate alone was Ln (C_0_/C) = 0.6906t—0.0702, R^2^ = 0.9826, while dimethoate plus TiO_2_/Ce was Ln (C_0_/C) = 0.7892 t + 0.1450, R^2^ = 0.9689 (Fig 1B). The half-life of dimethoate was 0.69 and 1.10 d with and without TiO_2_/Ce, respectively, illustrating TiO_2_/Ce could efficiently degrade dimethoate under field conditions. In addition, with TiO_2_/Ce hydrosol, ROS content in Bok choy was higher than the untreated controls (Fig 2). Under the sunlight, ROS generated by TiO_2_/Ce can accelerate the degradation of organophosphate insecticides. These combined results suggest that TiO_2_/Ce is a promising candidate for the photocatalytic degradation of pesticide residuals.

### Photocatalytic degradation products and pathway of dimethoate

**Fig 3:**
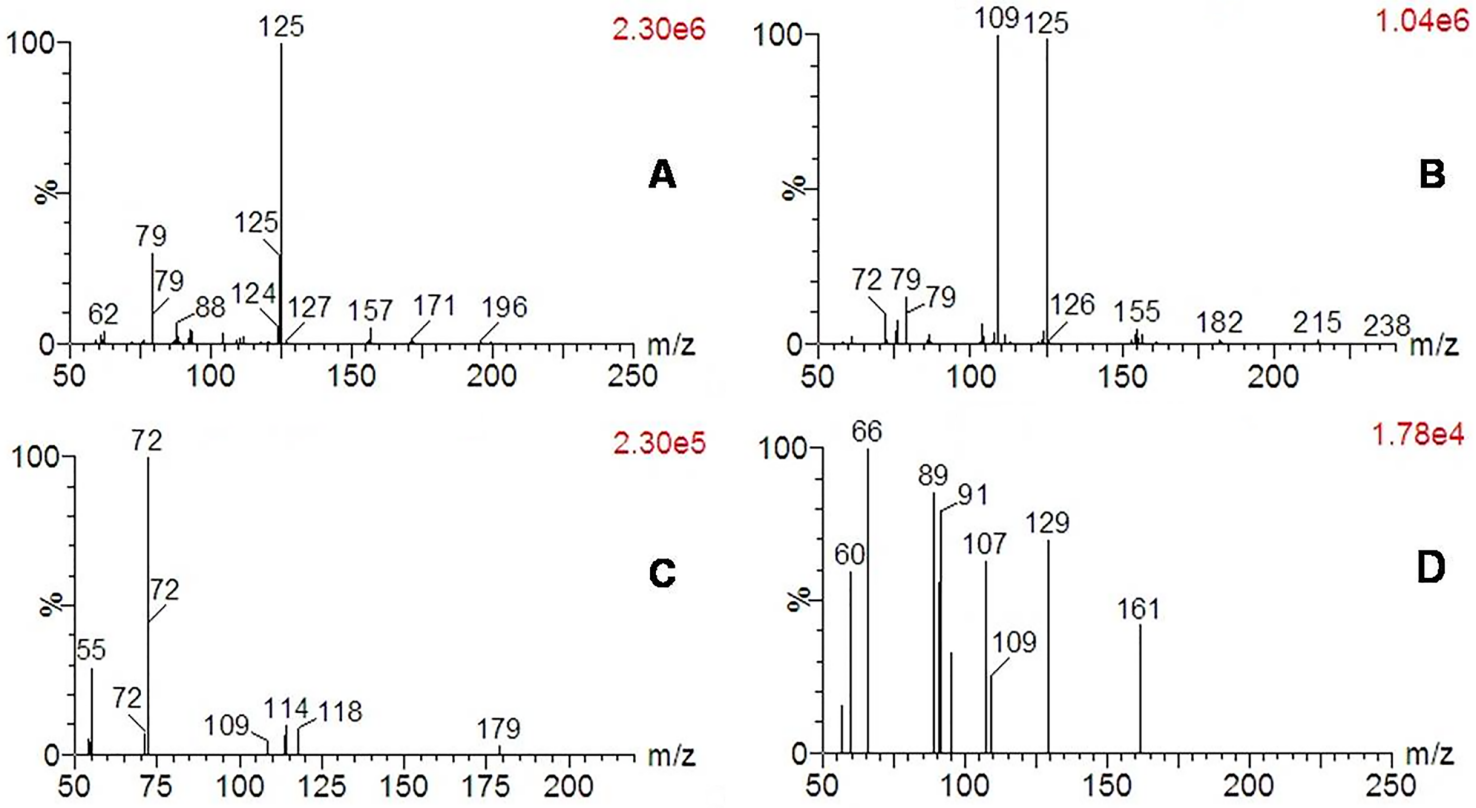
Mass spectrum of dimethoate and its degradation products in Bok choy in the presence of TiO_2_/Ce. **(A)** Dimethoate; **(B)** Product I omethoate; **(C)** Product II O,O,S-trimethyl thiophosphorothioate; and **(D)** Product III 1,2-Bis(acetyl-N-methyl-) methane disulfide.

The degradation products were identified by the analysis of mass spectrum. The mass spectrum of dimethoate and its photocatalytic degradation products are shown in Fig 3. In standard mass spectrums, the retention time of 4.245, 3.496, 4.182, 4.684 min and the their corresponding prominent protonated molecular ions at m/z = 230 [M + H]^+^, m/z = 214 [M + H]^+^, m/z = 195 [M + Na]^+^ and m/z = 209 [M + H]^+^ were found, and the compounds corresponding to the protonated molecular ions were identified as dimethoate (C_5_H_12_NO_3_PS_2_), omethoate (C_5_H_12_NO_4_PS), IUPAC name: 2-dimethoxyphosphorylsulfanyl-N-methylacetamide), O,O,S-trimethyl thiophosphorothioate (C_3_H_9_O_2_PS_2_) and 1,2-Bis (acetyl-N-methyl-) methane disulfide (C_6_H_12_N_2_O_2_S_2_). However, in the control samples, only dimethoate and omethoate were detected.

**Fig 4:**
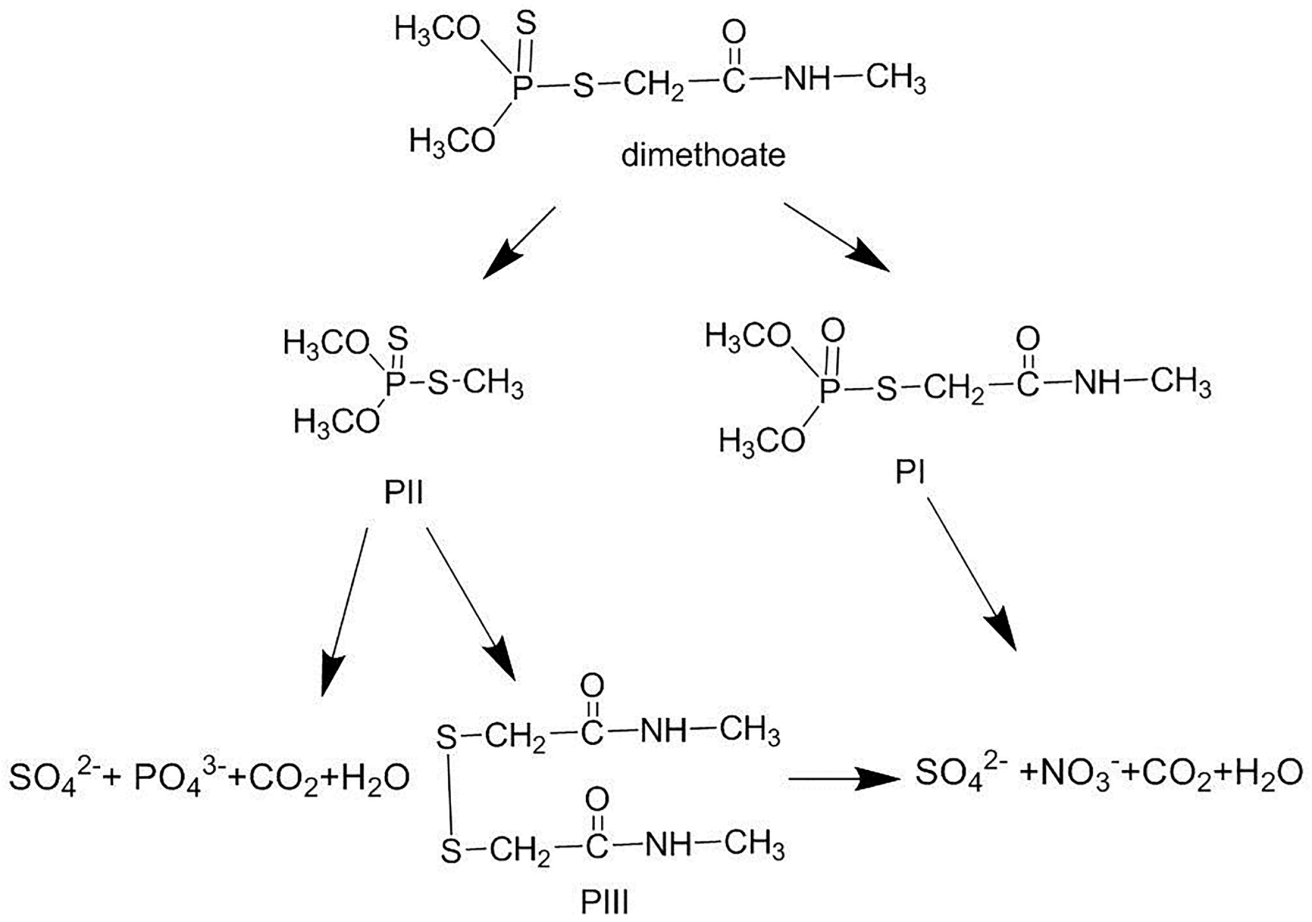
Proposed degradation pathway of dimethoate in Bok choy in the presence of TiO_2_/Ce. (PI) omethoate; (PII) O,O,S-trimethyl thiophosphorothioate; and (PIII) 1,2-Bis(acetyl-N-methyl-) methane disulfide.

According to the chemical structure of dimethoate and its metabolites, two pathways are proposed for dimethoate photodegradation (Fig 4). In the first proposed pathway, the sulphur atom in the P = S bond of dimethoate will be oxidized into P = O by the ROS attack, such as •OH radicals, which leads to the formation of omethoate. The formation of oxidized derivatives has been documented in the photocatalytic degradation of organophosphorus pesticides containing P = S group [28, 29]. Omethoate, a major byproduct of dimethoate decomposition [13, 30], could be subsequently mineralized into SO_4_^2-^, NO_3_^-^, CO_2_, and H_2_O through further oxidization and hydrolysis processes. In the second proposed pathway, the sulphur atom in the P-S or S-C bond could be attacked by positive holes to generate sulfide cation radicals to initiate the degradation reaction. This oxidation reaction process has been studied in the photocatalytic degradation of thiocarbamate pesticides and thioethers [31, 32]. Under the sunlight, photocatalytic reaction may produce a single electron from the sulfur atom, then initiate P-S or S–C cleavage. The cleavage of P–S bond forms •SCH_2_CONHCH_3_ radicals, and the cleavage of C–S bond forms (CH_3_O)_2_SPS• radicals. •SCH_2_C(O)NHCH_3_ radicals are easier to dimerize and lead to the formation of 1,2-Bis (acetyl-N-methyl-) methane disulfide [32]. Similarly, (CH_3_O)_2_SPS• radicals tend to combine with methyl radical (•CH_3_) to from the reaction media. O, O, S-trimethyl thiophosphorothioate will be generated after oxidization, and successively mineralized into SO_4_^2-^, PO_4_^3-^, CO_2_, and H_2_O.

### *Ab initio* quantum analysis of dimethoate degradation by TiO_2_ /Ce

**Fig 5:**
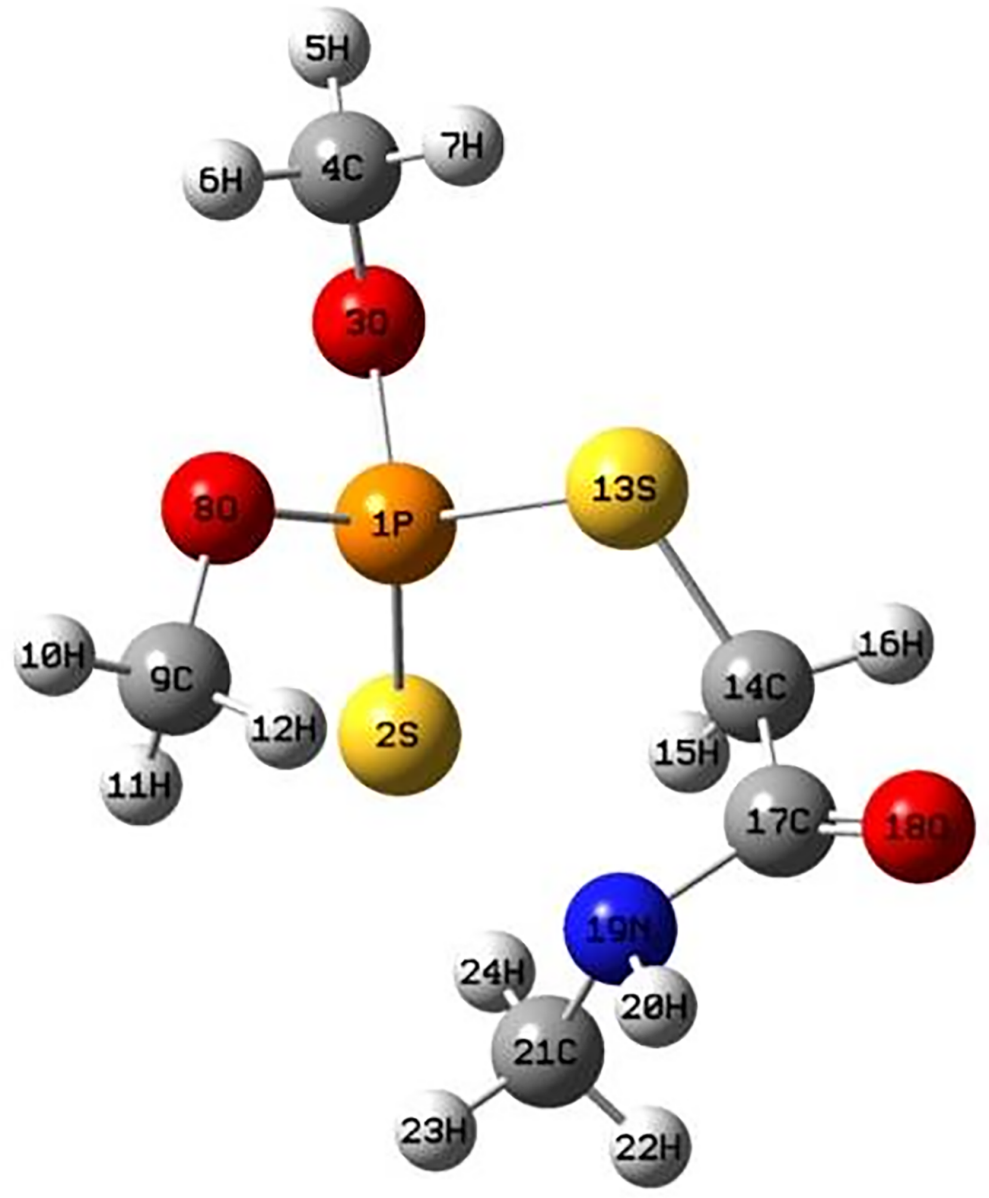
Spatial configuration of dimethoate molecule. This schematic drawing was based on the optimal geometry conformation at RHF/STO-3 level using the Gaussview3.08 graphic interface.

In order to further confirm the degradation products from UPLC-MS and reveal the degradation mechanism of dimethoate, quantum chemistry analyses of dimethoate were calculated based on the optimal geometry conformation of dimethoate molecule obtained at RHF/STO-3 level. The spatial configuration of dimethoate molecule is shown in Fig 5.

The bond lengths between atoms in dimethoate molecule are shown in Table 1. Specifically, the bond lengths between P^1^-S^13^, P^1^-S^2^ and S^13^-C^14^ are 2.1330×10^−10^, 1.99716×10^−10^, and 1.80856×10^−10^ m, respectively. The bond energy and length are negatively correlated [33]. The bonds between P^1^-S^13^, P^1^-S^2^ and S^13^-C^14^ are easier to cleave when attacked by ROS. When P^1^-S^2^ is attacked, P = S could be oxidized into P = O, which generates omethoate. If S^13^ is attacked, the bond S^13^-C^14^ could be cleavaged, which leads to the generation of O, O, S-trimethyl thiophosphorothioate.

**Table 1:** Bond length on main atoms in dimethoate molecule at the RHF/STO-3 level.

Bond	Bond length/ (×10^−10^ m)	Bond	Bond length/ (×10^−10^ m)
P^1^-S^2^	1.99716	P^1^- S^13^	2.13300
P^1^-O^3^	1.68445	S^13^- C^14^	1.80856
O^3^-C^4^	1.43702	C^14^- C^17^	1.54590
P^1^-O^8^	1.69091	C^17^- O^18^	1.21916
O^8^-C^9^	1.43474	C^17^- N^19^	1.45652

**Fig 6:**
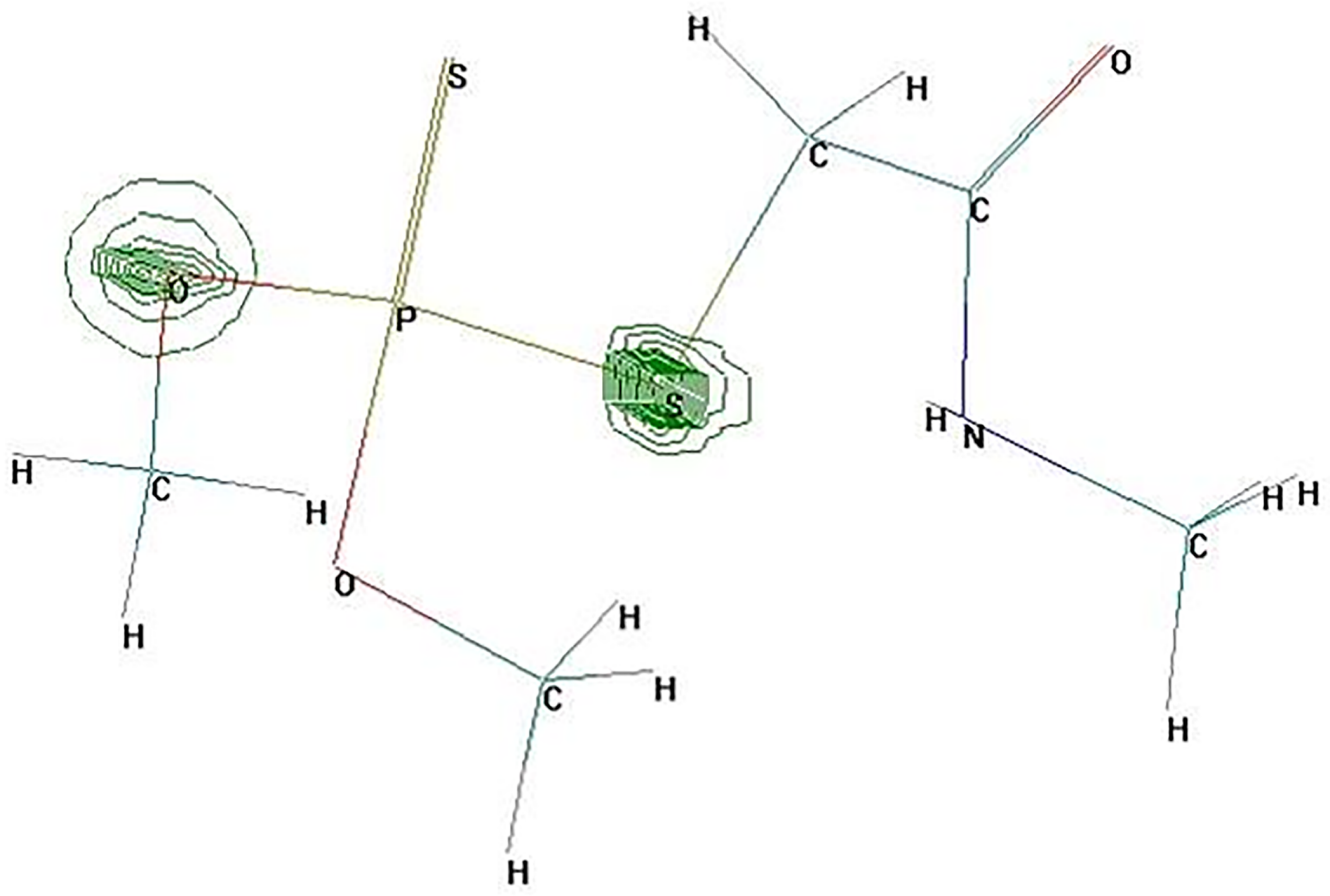
Atomic charge density of dimethoate molecule. This chart was generated using Hyperchem Release 7.0 program package.

Based on the Mulliken atomic charges of dimethoate (Table 2), the largest positive point charge of dimethoate molecule was located at P^1^ atom with a value of 0.779814, suggesting that the P^1^ atom is most likely attacked by nucleophilic reagents (e.g. H_2_O, OH^-^). The hydrolysis of phosphate ester bond leads to the generation of omethoate. This is a common process in the degradation of organophosphorus pesticides [34]. In addition, based on the total charge density of dimethoate (Fig 6), the atom S^13^ has the greatest electric charge density, demonstrating another vulnerable site of dimethoate. The existing S^13^ in P^1^-S^13^ and S^13^-C^14^ are the weaker sites under the attack by nucleophilic reagents, which is consistent with the results of bond length. Therefore, P^1^-S^13^ and S^13^-C^14^ are the likely initiation sites of photocatalytic degradation of dimethoate.

**Table 2:** Mulliken atomic charges of dimethoate molecule at the RHF/STO-3 level.

Atom	Charge	Atom	Charge
P^1^	0.779814	C^9^	-0.084473
S^2^	-0.333127	S^13^	0.036325
O^3^	-0.340566	C^14^	-0.226141
C^4^	-0.083176	C^17^	0.281797
O^8^	-0.352946	O^18^	-0.249928
N^19^	-0.3520827	C^21^	-0.106428

**Fig 7:**
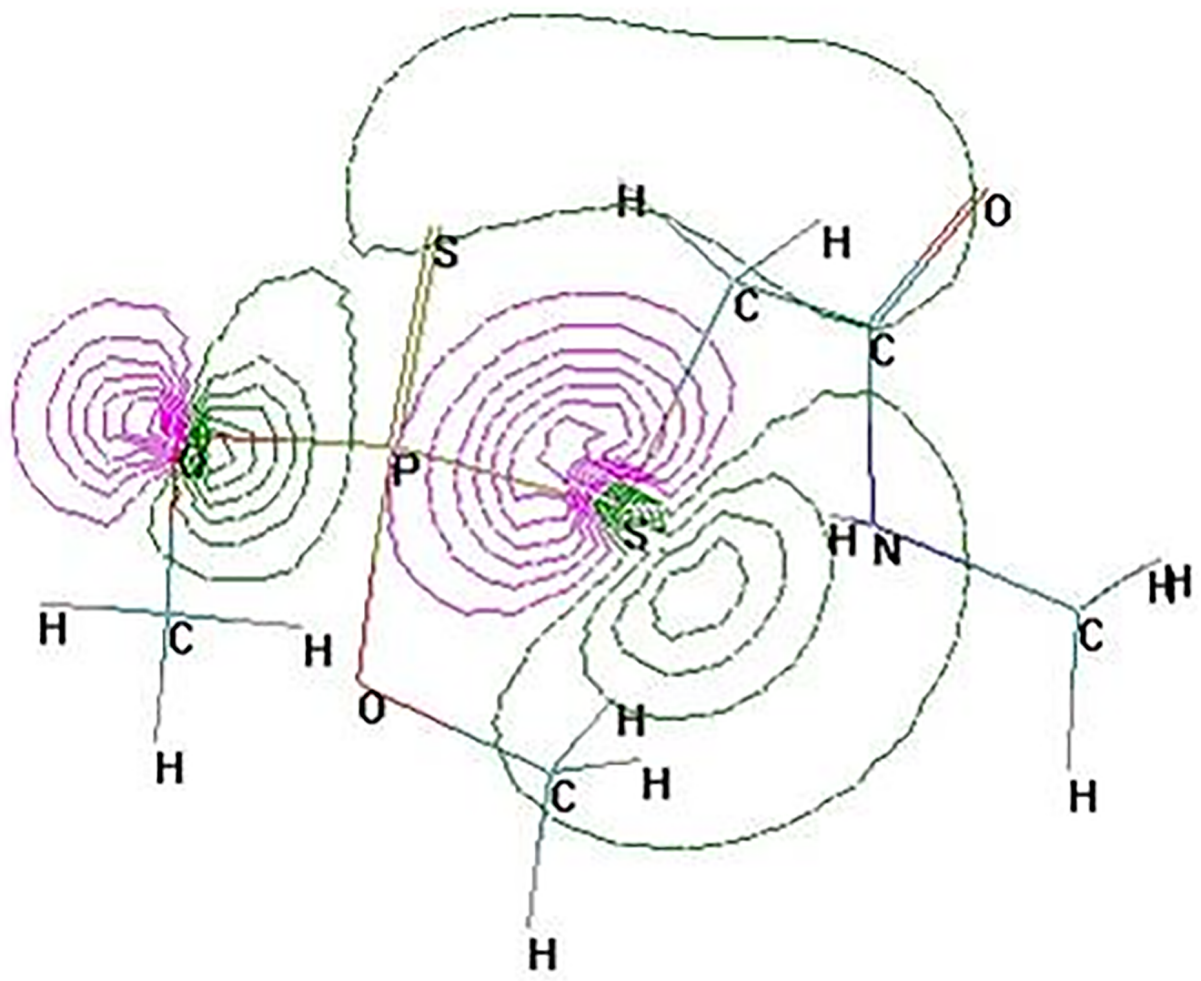
Frontier orbital density (HOMO) on main atoms of dimethoate molecule. This calculation was carried out using Hyperchem Release 7.0 program package.

Based on the frontier electron density (FED) value of the highest occupied molecular orbital (HOMO) in the dimethoate molecule, the atom S^2^ possesses the highest FED_HOMO_^2^ with the value of 1.020068 (Table 3). Additionally, the atom S^13^ has higher FED_HOMO_^2^ than other main atoms in dimethoate molecule (Fig 7). According to the frontier electron density theory, the atoms S^2^ and S^13^ are the vulnerable sites attacked by electrophilic reagent during the degradation of dimethoate. This theoretical research of frontier electron densities was reported by Parr and Yang [35]. Here, atom S^2^ readily extracts an electron to form dimethoate cation radical, and then P^1^ = S^2^ bond is cleaved, which allows P = S bond oxidize into P = O. This prediction confirms the formation of omethoate. Likewise, S^13^ releases an electron to form positive charged free radicals and then, is attacked by ROS to degrade dimethoate. Results from quantum chemistry analyses were consistent with the degradation products identified by UPLC–MS and proposed degradation pathway.

**Table 3:** Frontier electron densities (FEDHOMO2) on major atoms of dimethoate molecular at the RHF/STO-3 level.

Atom	FED_HOMO_^2^	Atom	FED_HOMO_^2^
P^1^	0.008667	S^13^	0.084516
S^2^	1.020068	C^14^	0.017092
O^3^	0.019753	C^17^	0.000117
C^4^	0.001158	O^18^	0.002393
O^8^	0.003233	N^19^	0.001243
C^9^	0.000307	C^21^	0.011392

## Discussion

### Degradation efficiency of dimethoate using nano-TiO_2_

Nanoparticles have been well accepted in environment, pharmaceutical and food industry [36–39]. As an attractive material, nano-TiO_2_ is widely used in many fields. In recent decade, nano-TiO_2_ plays an important role in sustainable agriculture, concerning the improvement of nutrient use efficiency [40], management of the diseases and enhancement of crop yields [41]. However, there has been relatively little applications in the degradation of pesticide residues in field trials. Zeng et al. demonstrated the rare-earth doped nano-TiO_2_ can increase the degradation efficiency of chlorpyrifos, acephate and carbendazim residues in tomato leaves and soil under the sunlight [42]. Liu et al. demonstrated that the degradation of acephate in pak choi was significantly accelerated in the presence of TiO_2_/Ce [29]. In addition, Evgenidou et al. found the photocatalytic degradation reaction of dimethoate using TiO_2_ was a first-order process [43]. These combined reports suggest that TiO_2_ can speed up the degradation of pesticide residuals.

Here, the application of TiO_2_/Ce indeed led to a rapid degradation of dimethoate residue in Bok choy. With TiO_2_/Ce, the half-life of dimethoate is 1.6 times shorter, which means the pre-harvest interval between dimethoate application and harvest will be significantly reduced. The recommended intervals for dimethoate application on vegetables are, at least, 10 days before the harvest. In practice, however, excessive use of pesticides and the ignorance of the recommended intervals often results in vegetables containing pesticide residues exceeding the maximum limits established by FAO/WHO. The rapid removal of pesticide residues by TiO_2_/Ce might be a potential resolution. Specifically, TiO_2_/Ce can be applied on targeted vegetables 3-days prior to the harvest. Although there are some concerns regarding the potential phytotoxicityto Bok choy as a result of the ROS burst generated from the exposure to TiO_2_/Ce [44], we did not observe any adverse effects in our field trials, which is consistent with Larue et al [45].

TiO_2_ is, in general, considered nontoxic. The plant growth and yield can be promoted by higher concentrations of nanoscale TiO_2_ [46, 47]. There is no damage caused by the consumption of farm products sprayed with TiO_2_ as a photocatalyst [48]. However, previous studies have demonstrated acute toxicity of nanoscale TiO_2_ to mice [49]. Although low dose TiO_2_ had negligible impacts on haemostasis blood system and immune system in mice after oral intake, Duan et al. noted that higher dose can damage liver function [50]. In addition, higher dose of TiO_2_ can have negative impacts on aquatic ecosystems [51] and soil microbiota [52]. Therefore, assessing the environmental risks of TiO_2_/Ce nanoparticle and examining the responses of mammalian species to vegetables containing nano- TiO_2_/Ce are warranted before the adoption of this technology in agricultural practices.

### The degradation products and possible pathway of dimethoate

TiO_2_ mediated photocatalytic degradation of pesticides has proven to be a promising method to purify the polluntants from the environment. Nano-TiO_2_ are capable of degrading dimethoate has been reported [53–55]. However, the degradation pathway of dimethoate has only been reported in a slurry system with TiO_2_ [56]. To date, there is no clear photocatalytic degradation pathway of dimethoate reported in any crops by TiO_2_ in field trials. In the present study, TiO_2_/Ce hydrosol could efficiently degrade dimethoate residue in Bok choy under field conditions, suggesting that TiO_2_/Ce is a promising candidate for the photocatalytic degradation of pesticide residues of plants in agricultural production. Due to the fast mineralization capability of TiO_2_ and simple structure of dimethoate, only three degradation products, omethoate, O, O, S—trimethyl thiophosphorothioate and 1, 2—Bis (acetyl—N—methyl -) methane disulfide, were successfully confirmed with UPLC-MS analysis combined with the quantum chemistry analysis. According to the dimethoate and its intermediates, two degradation pathways of dimethoate by TiO_2_/Ce in Bok choy are proposed for the first time. Additionally, the degradation of dimethoate has elucidated in microbes and in mammals. In microbial degradation of dimethoate, employing the *Pseudomonas aeruginosa* of bacterial strain, four unknown degradation products were detected by thin layer chromatography [57]. However, degradation pathway of dimethoate was not put forward. In mammals, dimethoate could be desulfurated to form omethoate through cytochrome P450s and was subsequently metabolized into dimethoate carboxylic acid, or could be directly hydrolysised to form dimethoate carboxylic acid through esterase-dependent hydrolytic cleavage of the C-N bond. Dimethoate carboxylic acid was then metabolized further into non-toxic metabolites, including dimethyldithiophosphate, dimethylthiophosphate and dimethylphosphate [58].

As regard to the mineralization of dimethoate, results are inconsistent. Evgenidou et al demonstrated TiO_2_ was not able to mineralize dimethoate [43]. Dimethoate in water achieved the 100% decomposition using TiO_2_ immobilized on silica gel under UV exposure, however, it was not completely mineralized [59]. When wastewater containing dimethoate was processed by photocatalysis–biological coupled system, dimethoate was totally decomposed with 90% mineralization and complete nitrification [60]. The mineralization by-products of dimethoate warrant further investigation to confirm the final products of pesticide decomposition are harmless inorganic ions and to determine which photocatalytic process should applied to alleviate the pesticide residual issue.

### Photodegradation and molecular structural of dimethoate

Degradation of organophosphorus pesticide mainly involves hydrolysis and photodegradation. A primary mechanism of hydrolysis is the substitution reaction of nucleophilic group attacked by H_2_O and OH^-^. Photodegradation is a process of the chemical bond cleavage of molecules excited by lights. The former is a process associated with positive charge of atoms, and the latter is a process related to the molecular bond length, atomic charges and other quantum parameters. In principle, the bond length, atomic charge and frontier electron density of the highest occupied molecular orbital are positively correlated with the degradation activity. The degradation mechanism proposed in this study is testified and improved by the *ab initio* quantum chemistry. Sun et al. performed a study on degradation mechanism of polychlorinated dibenzo-p-dioxins using high-level molecular orbital theory calculations [61]. Zhang et al. employed Gaussian 03 package to investigate the atmospheric photooxidation of dichlorvos [62]. In this study, the resultant degradation pathways are energetically feasible for dimethoate photodegradation by TiO_2_/Ce in Bok choy and are consistent with the three degradation products we observed empirically.

## Conclusion

TiO_2_/Ce hydrosol can accelerate the degradation of dimethoate in Bok choy under the field conditions, especially within 3 d of photocatalytic treatment. ELISA results suggest that ROS generated by TiO_2_/Ce facilitates the rapid degradation. Three degradation products of dimethoate, including omethoate, O,O,S-trimethyl thiophosphorothioate, and 1,2-Bis (acetyl-N-methyl-) methane disulfide are detected by UPLC-MS. In addition, two pathways are proposed for dimethoate photodegradation, which are confirmed by *ab initio* quantum chemistry analyses. Our combined results indicate that photocatalytic degradation of pesticides using TiO_2_/Ce is a feasible alternative to managing pesticide residues in vegetables.
